# Spatial Differentiation Characteristics and Evaluation of Cu and Cd in Paddy Soil around a Copper Smelter

**DOI:** 10.3390/toxics11080647

**Published:** 2023-07-26

**Authors:** Yuan Ding, Li Xi, Yujing Wu, Yihong Chen, Xiaoping Guo, Hong Shi, Shuo Cai

**Affiliations:** 1 National-Local Joint Engineering Research Center of Heavy Metals Pollutants Control and Resource Utilization, Nanchang Hangkong University, Nanchang 330063, China; Xili99050802@163.com (L.X.); 18307918809@163.com (Y.W.); 17779549577@163.com (Y.C.); 13607993963@163.com (X.G.); 17370836276@126.com (S.C.); 2 College of Environment and Chemical Engineering, Nanchang Hangkong University, Nanchang 330063, China; 3 Jiangxi Key Laboratory of Agricultural Efficient Water-Saving and Non-Point Source Pollution Preventing, Jiangxi Central Station of Irrigation Experiment, Nanchang 330063, China

**Keywords:** copper smelter, potentially toxic elements (PTEs), migration rate (M_R_), speciation, soil colloid, iron oxides

## Abstract

To accurately evaluate the distribution and bioavailability of potentially toxic elements (PTEs) such as Cu and Cd in farmlands near a copper smelter, we determined the total concentrations (Cu-T and Cd-T), various speciation concentrations of Cu and Cd and physicochemical properties of 18 paddy soil (or colloid) samples in Guixi town, Jiangxi province, China. The results showed that the concentrations of Cu-T and Cd-T in the soil around the smelter far exceeded the standard limits. Specifically, Cu ranged from 97.47 to 1294.63 mg·kg^−1^, with a coefficient of variation (CV) of 0.95; Cd ranged from 0.14 to 9.06 mg·kg^−1^, and the CV was 1.68. Furthermore, the pollution of PTEs continued to accumulate, posing a significant risk to the environment and human health. The findings from the analysis of soil and colloid indicated that the distribution characteristics of Cu and Cd speciations did not align with the total concentrations. The highest pollution points were found to be shifted to the residual fraction of Cu, organic fraction, and crystalline iron oxide fraction of Cd in soil. The dominant fraction of Cu in colloid was the amorphous iron oxide fraction, whereas Cd was the crystalline iron oxide fraction. The assessment of Cu and Cd migration (M_R_) revealed that Cd posed a greater ecological risk. Further examination of the properties of iron oxides in soil and colloid revealed that they played a crucial role in the migration and transformation of soil PTEs.

## 1. Introduction

Copper smelting is an industry notorious for its heavy pollution, characterized by the emission of atmospheric particulates and other waste materials containing heavy metals such as Cu, Cd, Pb, and Zn [1]. Research findings indicated that Cu and Cd were two of the primary pollutants in the soil surrounding the copper smelter [2]. These two elements are classified as potential toxic elements (PTEs), known for their highly toxic and non-degradable properties [3]. Moreover, they can be transferred to the human body through the food chain, and their toxicity can result in bioaccumulation or biomagnification, which can lead to many diseases, and are poisonous, mutagenic, and carcinogenic to humans [4,5].

The assessment of contaminated soil is typically based on total concentrations of potentially toxic elements (PTEs) because of the ease of monitoring and lower cost compared to other methods [6]. In response to the multi-element co-contamination issues surrounding copper enterprises, evaluation indexes are often used to comprehensively assess the pollution situation of multiple elements. Evaluation indexes, such as EF (enrichment factor) [7], IPI (integrated pollution index) [8], and I_geo_ (geo-accumulation index) [9], provide effective methods for evaluating the pollution degree of PTEs in soil. By comparing the observed data of PTEs with the background values, I_geo_ produces a concise score to reflect the pollution condition [10]. This approach has been widely adopted, as it enhances our comprehension of the extent of associated PTEs’ pollution [11].

While most evaluation index methods based on total concentrations can indicate soil pollution to a certain extent, they don’t account for the bioavailability and potential risk of PTEs [12]. The bioavailability, migration, and transformation of PTEs in soil are mainly affected by the physicochemical properties of soil, such as pH, redox potential, iron oxides, and organic matter, except for the total concentrations of PTEs [10]. Research by Liu showed that iron oxides in soil significantly affected the speciation of PTEs [13]. PTEs were transformed into more stable speciations during the process of iron species transformation and iron redox mediated by iron-reducing bacteria [14]. Chemical extraction methods, such as the Tessier extraction method, have been widely accepted by researchers to express the distribution characteristics and bioavailability of Cu and Cd in soil [15]. To explore the effects of crystalline and amorphous iron oxides on the activities of Cu and Cd, the modified Tessier extraction method was adopted in this paper. The speciation of Cu and Cd in soil was divided into the exchangeable and the carbonate fraction (F1), readily reducible iron and manganese oxide fraction (F2), organic fraction (F3), crystalline iron oxide fraction (F4), and residual fraction (F5) [16], with decreasing bioavailability of Cu and Cd in soil. To accurately depict the intricate dynamics of heavy metal redistribution and fixation in soil, Han and Banin [17] devised a novel evaluation method, the reduced partition index (I_R_). This approach was based on a sequential selective dissolution procedure, enabling the assessment of heavy metal fixation in soil. I_R_ represented the weighted sum of each heavy metal fraction, with the weight of each fraction gradually increasing as its activity decreased. In the pursuit of tracing the heavy metal fractions loosely bound to the soil, Burachevskaya [18] employed Miller’s method as a sequential selective extraction technique combined with I_R_, and effectively gauged the levels of Cu, Pb, and Zn pollution surrounding a coal-fired power plant. It provided a comprehensive understanding of the redistribution and fixation characteristics of heavy metals in soil. Conversely, to conveniently compare the activity and migration degree of Cu and Cd in the soil at different sites and highlight the influence of the exchangeable and carbonate fractions, readily reducible iron and manganese oxide fractions, the modified weight calculation method based on I_R_ was used in this paper to quantitatively calculate the migration of Cu and Cd in soil—migration rate M_R_. However, the weights of each fraction in this method decrease gradually with the decrease in activity.

Guixi town in Jiangxi province, China, is home to the most advanced and largest flash copper smelter in Asia. Early research results showed that the total concentrations of Cu and Cd in the paddy soil under the comprehensive slag field dam of the smelter ranged from 102.31 to 415.54 mg·kg^−1^ and from 0.33 to 6.87 mg·kg^−1^, respectively (MEEP, 2018b Cu < 50 mg·kg^−1^, Cd < 0.3 mg·kg^−1^) [19,20]. Moreover, survey results on paddy soil in the village of Shuiquan and Zhushan showed that concentrations of Cu and Cd were from 6.17 to 11.7 and from 3.02 to 3.41 times as much as the background, respectively. The tested villages were located in the southwest direction of the copper smelters, which was the annual downwind direction [21]. This copper smelter has both non-point and point source pollution characteristics, resulting in soil quality that is worse than the farmland standard. While non-point source pollution caused by the slag field dam has been effectively addressed [22], the long-term and positioned emission of soot from the smelter would continue to increase the concentrations of Cu and Cd in the soil as point source pollutions [23]. Therefore, further investigation and analysis of PTEs’ pollution around the smelter are necessary.

As mentioned above, the bioavailability and migration of Cu and Cd in soil not only depends on the total concentrations of PTEs but are also affected by the physicochemical properties of the soil. This paper focuses on the paddy soils surrounding a typical copper smelter in southern China and aims to analyze: (1) the current situation of soil Cu and Cd pollution around the smelter on a small scale; (2) the distribution characteristics of various Cu and Cd species in southern China polluted by non-point sources and atmospheric point sources; and (3) the spatial heterogeneity and bioavailable evaluation of Cu and Cd distribution in this region.

## 2. Materials and Methods

### 2.1. Study Site

The copper smelter was situated in the northeastern region of Jiangxi Province, located within the intermediary zone between the Wuyi Mountain and the Poyang Lake Plain. The climatic conditions of the area are characterized by a subtropical monsoon climate, which is marked by high temperatures, ample sunshine, and copious rainfall. The principal wind direction in the region is easterly, with northeasterly winds being the secondary dominant direction throughout the year.

The copper smelter under study has adopted the world’s most advanced oxygen-enriched flash smelting technology and double contact double adsorption acid production technology. The primary pollutants discharged from the plant are PTEs, such as Cu and Cd.

### 2.2. Soil Sampling

**Figure 1:**
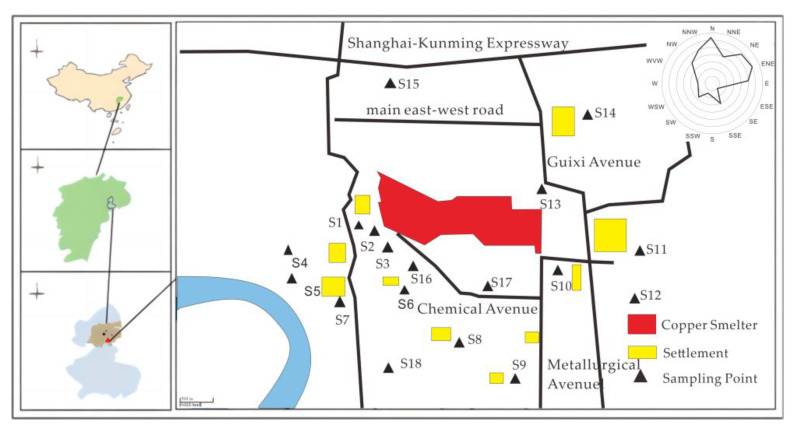
Map of sampling points.

In November 2020, we collected soil samples from 18 sites in the farmland surrounding the copper smelter, from the surface layer (0–20 cm). The selection of sampling sites adhered to the fan-type distribution principle of the atmospheric point source, as prescribed by “the Technical Specification for Soil Environmental Monitoring (HJT 166–2004)” published by the Ministry of Ecology and Environment of the People’s Republic of China (MEEP 2004) [24]. To account for the pollution characteristics in the smelter area, we intensified the sampling frequency in the non-point source region, as shown in Figure 1.

Three sampling points were selected from each site, with a minimum distance of 1 m between them. About 500–750 g of samples were collected from each point and mixed to get a composite sample. Before sample collection, extraneous materials such as small gravel, waste plastics, and dead branches were removed from the sampling area. The samples were air-dried in the laboratory, ground, and passed through a nylon sieve with a 2 mm aperture before being sealed for later use.

### 2.3. Sample Analysis

The physicochemical properties of the soil were analyzed according to the method of Bao [25]. The soil pH was measured using a pH meter (PHS-3C, Shanghai) at a water-to-soil ratio of 2.5:1 (V/m). Soil organic matter (SOM) was determined using the K_2_Cr_2_O_7_ oxidation method. Available phosphorus (AP) was extracted by 0.5 mol·L^−1^ NaHCO_3_ (soil-to-water ratio of 20:1) and measured using the molybdenum–antimony colorimetric method. Free iron (Fe_d_) in the soil, which includes amorphous ferric oxide (Fe_o_) and crystalline ferric oxide (Fe_c_), was extracted according to the method of Zhang [26]. The total concentrations of Cu and Cd (Cu-T and Cd-T) in the soil samples were determined using ICP-MS after digestion with HCl–HNO_3_–HClO_4_.

To investigate the effects of soil iron oxides on the migration and transformation of PTEs, different fractions of Cu and Cd in the soil samples were sequentially extracted using a modified Tessier method to determine the distribution of Cu and Cd speciations around the smelter (Table 1) [16]. This method allowed for the identification of Cu and Cd speciation in the exchangeable and carbonate fraction (F1), readily reducible iron and manganese oxide fraction (F2), organic fraction (F3), crystalline iron oxide fraction (F4), and residual fraction (F5).

Liu [27] mentioned the preparation of soil colloid, where the heavy metal fractions present in the soil colloid can be classified into three distinct species, namely the amorphous iron oxide fraction, the crystalline iron oxide fraction, and the residual fraction [16].

### 2.4. Methods of Pollution Assessment

#### 2.4.1. Geo-Accumulation Index

The index of geo-accumulation (I_geo_) is used to assess soil contamination. I_geo_ is calculated by the following equation [28]:

I_geo_ = log_2_[C_i_/1.5B_n_] (1)

where C_i_ is the concentration of the examined metal in the soil or sediment, B_n_ is the geochemical background value of the metal in the equation in the shale, and factor 1.5 accounts for the possible variations in the background values.

The I_geo_ scale consists of seven grades, as shown in Table 2.

#### 2.4.2. Migration Rate (M_R_)

We used M_R_ to quantify the distribution and migration characteristics of Cu and Cd, and M_R_ is calculated by the following equation [18]:

(2) $$M_{R}=\frac{\sum_{i=1}^{k}\left(F_{i}\times {\left(k-i+1\right)}^{n}\right)}{k^{n}}$$

where, F is the percentage content of each fraction of PTEs; i is the extraction step number; k is the number of total extraction steps and equals 5 in this paper; n is set to 2 in this paper.

### 2.5. Data Analysis

The statistical analysis, including normal distribution testing and correlation analysis, was performed using IBM SPSS Statistics 26.0 software. The figures were generated by Origin 2021. The spatial distribution characteristics maps of PTEs’ pollution were generated by ArcMap 10.5.

## 3. Results

### 3.1. Characteristics of the Soil around a Copper Smelter

The physicochemical properties of the soil around the copper smelter are displayed in Table 3. The soil pH ranged from 4.04 to 5.16, with a coefficient of variation (CV) of 0.06, which was typical of soil acidity in the middle and lower branches of the Yangtze River, China. The concentrations of SOM in the study area showed an approximately normal distribution, with 66.67% of samples meeting the third-level standard, while the others were lower than the middle level, according to the classification standard of soil nutrients in the Second National Soil Survey of China [29]. The concentrations of AP showed an approximately log-normal distribution, with 83% of samples meeting level 3 or greater than the standard. Furthermore, the total iron (Fe-T) concentrations in the soil met level 2 or greater than the standard. Therefore, there was no nutrient stress in this region, and the impact of nutrient stress on soil PTEs would not be considered below.

The average concentrations of Cu and Cd in the study area were higher than the local background levels by 15.65 and 10.56 times, respectively [21], and by 6.35 and 3.8 times the soil heavy metal pollution risk screening value (Table S1) [20], suggesting that Cu and Cd were severely polluting the study area. The distribution characteristics of Cu and Cd demonstrated that the CVs were 0.95 and 1.68, respectively, indicating that the heterogeneity of soil heavy metals in the study area was significant, which was consistent with industrial areas such as smelters.

### 3.2. Distribution and Evaluation of the Total PTEs

The concentrations of Cu and Cd in the soil in the study area ranged from 97.47–1294.63 mg·kg^−1^ and 0.14–9.06 mg·kg^−1^, respectively. Wang [30] previously investigated the region and reported that the concentrations of Cu and Cd in the vicinity of the smelter were 38.14–586.21 mg·kg^−1^ and 0.48–3.79 mg·kg^−1^, respectively. These results indicate that the emission of the smelter has led to a continued accumulation of PTEs’ pollution in the area, necessitating further investigation.

**Figure 2:**
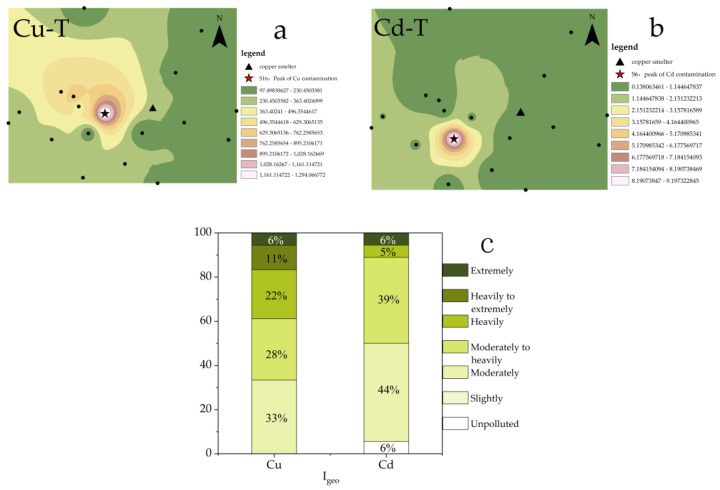
(**a**). Spatial distribution of Cu-T; (**b**). Spatial distribution of Cd-T; (**c**). Evaluation of Cu and Cd.

The distributions of PTEs in the study area were assessed using Cu-T and Cd-T (Figure 2a,b). The maximum concentrations of both Cu and Cd were found to occur in the downwind region (west) of the local smelter, with the highest pollution points being S16 and S6, respectively. These findings were consistent with previous investigations into the spatial distributions of Cu and Cd in the soil of the region [31] and confirmed that the heterogeneity of Cu and Cd distribution was caused by the complex pollution sources and the different migration characteristics of Cu and Cd [32].

To visually evaluate the accumulation of Cu and Cd in the study area, I_geo_ was utilized (Figure 2c). The results indicated that all sampling points in the study area were above moderately polluted for Cu, with approximately 39% of points classified as heavily polluted or worse. In contrast, while some sampling points for Cd were unpolluted, most (89%) were classified as moderately to heavily polluted. In summary, the pollution of Cu in the study area was found to be more severe than Cd.

### 3.3. Spatial Distribution of PTEs’ Speciations

**Figure 3:**
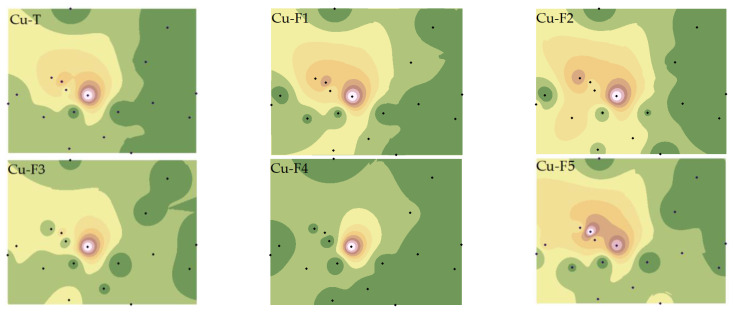
Spatial distributions of Cu total and fraction concentrations. Note: Exchangeable and carbonate fractions (F1), readily reducible iron and manganese oxide fractions (F2), organic fractions (F3), crystalline iron oxide fractions (F4), residual fractions (F5).

**Figure 4:**
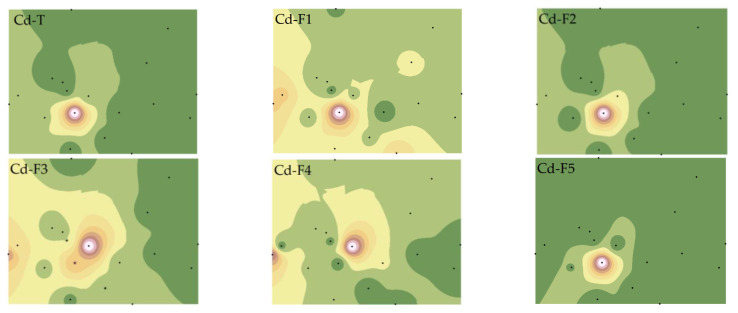
Spatial distributions of Cd total and fraction concentrations. Note: Exchangeable and carbonate fractions (F1), readily reducible iron and manganese oxide fractions (F2), organic fractions (F3), crystalline iron oxide fractions (F4), residual fractions (F5).

We have refined the Tessier method (2.3 Materials and Method) to comprehensively consider the effects of F2 and F4 on the bioavailability of Cu and Cd and have analyzed the spatial distribution characteristics of various PTE speciations (Figure 3 and Figure 4).

As depicted in Figure 3, the western region of the smelter was most severely affected by Cu pollution, with pollution severity increasing closer to the smelter. The distribution characteristics of Cu speciations were distinct from Cu-T, with the highest pollution point of Cu-F5 varying, while the highest pollution point and highest secondary pollution point of Cu-F1, Cu-F2, and Cu-F5 coexisted. This phenomenon may be attributed to the differences in iron oxides and pH in the study area, which significantly impact the spatial distribution characteristics of Cu [33,34].

The spatial distributions of Cd-T and various fractions differed from Cu. Except for Cd-F2 and Cd-F5, which showed high similarity with Cd-T, the others were quite distinct, particularly the significant variation of Cd-F3 and Cd-F4 [35]. This suggests that Cu and Cd in soil may have different sources, or distinct migration characteristics due to the reactions between PTEs and other materials (such as iron oxides, SOM, AP, etc.) in the soil [36].

Further statistical analysis of soil Cu and Cd speciations (Table S2) revealed that the CVs of Cu and Cd speciations ranged from 0.95 to 1.44 and from 0.86 to 2.65, respectively, indicating that the spatial heterogeneity of Cd in the study area was higher and greatly influenced by human factors. Cu was primarily present in non-residual fractions, with the content of Cu-F4 being relatively low (2.63–14.30%) (Table S1). The primary fractions of Cd were F1 (18–74%) and F5 (11–73%) (Table S1), confirming that the non-active components of the two elements existed differently, and that the total concentration could not fully express the bioavailability of Cu and Cd.

### 3.4. Distribution of PTEs in Soil Colloid

**Figure 5:**
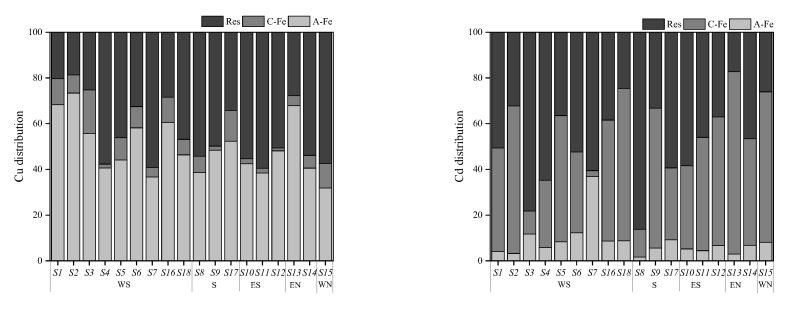
Distribution of each form of Cu and Cd in soil colloids. Note: A-Fe: amorphous iron oxide bound fraction; C-Fe: crystalline iron oxide bound fraction; Res: residual fraction.

A conspicuous difference was observed between the distributions of Cu and Cd speciations in the investigated area, which was influenced by soil physicochemical properties such as pH, AP, SOM, and iron oxides, among others [37,38]. As is well-known, soil colloid boasts a vast specific surface area and carries negative charges, thereby playing an important role in the distribution of PTE speciations and determining the concentration ratio and composition of amorphous and crystalline iron oxide fractions of PTEs in soil [39]. Consequently, this paper focused on the distribution of PTEs speciations in soil colloid, and the results are presented in Figure 5.

The concentrations of Cu and Cd in soil colloid ranged from 185.45 to 1727.15 mg·kg^−1^ and from 0.25 to 9.95 mg·kg^−1^, respectively, which were significantly higher than those in soil, underscoring the fact that soil colloid served as the main carrier of PTEs in soil. Cu in colloid was dominated by the amorphous iron oxide fraction (32–73%) and residual fraction (20–59%), whereas the main occurrence fractions of Cd were the residual fraction (17–86%) and crystalline iron oxide fraction (10–80%). This could be one of the principal reasons for the spatial heterogeneity of Cu and Cd distribution.

## 4. Discussion

### 4.1. Factors Affecting the Spatial Heterogeneity of Soil PTEs

**Figure 6:**
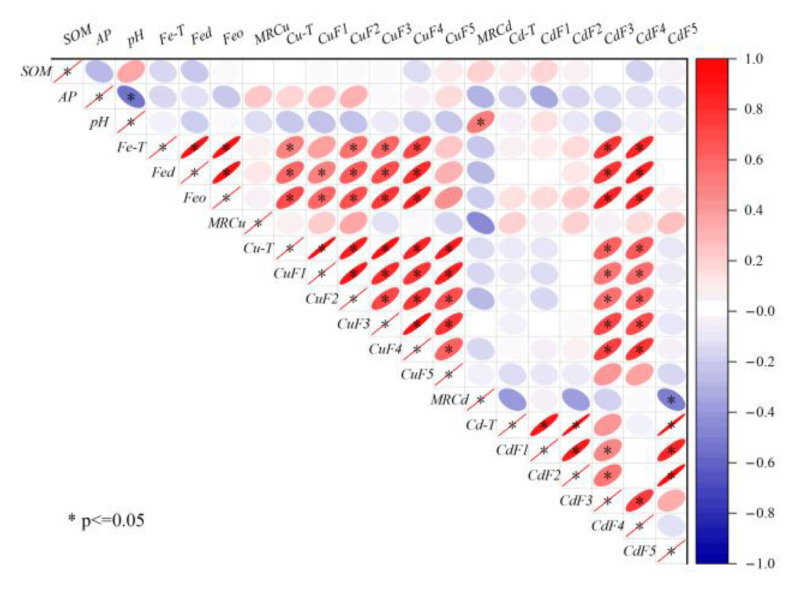
Correlation among Cu-T, Cd-T, fraction concentrations, and soil basic properties.

The present study utilized correlation analysis to investigate the impact of soil physicochemical properties on the distribution of Cu and Cd speciations in the farmlands surrounding the smelter. The results, as depicted in Figure 6, demonstrate that soil physicochemical properties, such as SOM, AP, and pH, did not exhibit any significant correlation with Cu and Cd. This finding was consistent with the characteristics of the area with continuous external pollution [40]. However, iron oxides (Fe-T, Fe_d_, and Fe_o_) showed a positive correlation with the non-residual Cu, Cd-F3, and Cd-F4, indicating that soil iron oxides were the main factors regulating the migration and transformation of PTEs [41]. Previous studies have established that iron oxides were the primary carriers of PTEs, which influenced the bioavailability of PTEs [42]. Iron oxides mainly retain PTEs through surface complexation, surface precipitation, and structural incorporation [43]. In combination with Figure 5, it was observed that Cu had a high affinity with Feo, and unlike Cu, Cd has a high affinity with Fec. This finding was a testament to the fact that the species of iron oxide significantly affected the binding, distribution, and bioavailability of PTEs [44].

Cu-T exhibited a positive correlation with the speciations, with Cu-F1 displaying the highest correlation, which was consistent with the spatial distribution of Cu-T. On the other hand, a significant correlation was observed between Cd-T and Cd-F5, whereas the correlation between Cd-T and Cd-F3 (or Cd-F5) was not significant, similar to their spatial distribution. This observation further confirms that total concentrations cannot entirely describe the migration and transformation of PTEs. The properties of PTEs were also one of the main factors affecting their distribution [45]. Cu and Cd were significantly correlated only with F3 and F4, and also with iron oxides, which proved that the interaction between iron oxides and SOM had an important influence on the regulation of the migration and transformation of PTEs [46].

### 4.2. Assessment of PTE Pollution around the Smelter

This study assessed the pollution characteristics of the study area, with a focus on two main pollutants, namely Cu and Cd, and their respective spatial distribution characteristics. Based on the analysis of total concentrations, it was observed that the pollution of Cu around the smelter was more severe than Cd (Figure 2c). However, research indicates that the ecological risk and toxicity of Cd are higher than Cu, owing to its high bioavailability and mobility [47]. To assess the risk of PTE pollution, conventional evaluation methods typically involve weighting the toxicity coefficient of elements based on their total concentrations, for example, the Ecological Risk Index (RI) [48].

**Figure 7:**
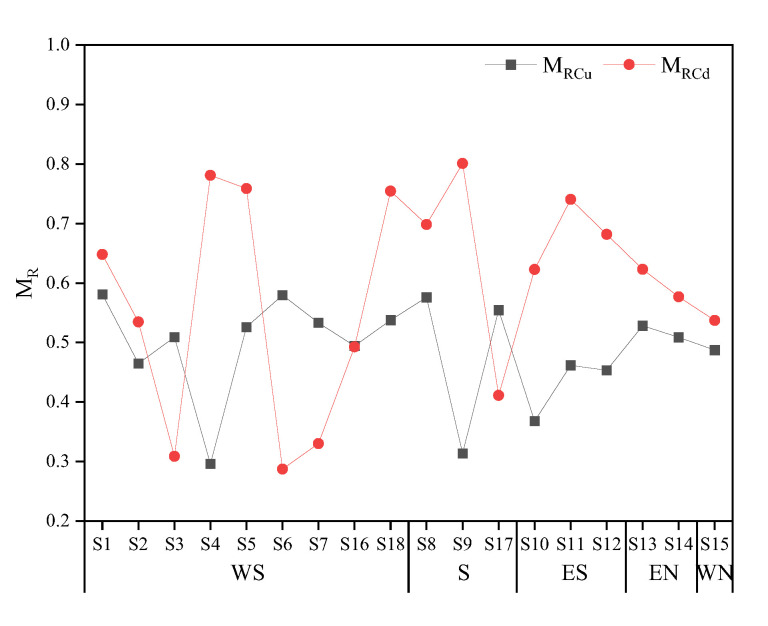
M_R_ of Cu and Cd in the study area.

Based on speciation analysis, this paper employed a weighted summation method to calculate the migration (M_R_) of PTEs, to emphasize the role of F1, F2, F3, and other active fractions in the migration. The calculation results are presented in Figure 7. The higher the M_R_ value, the higher the potential risk. The M_R_ value served to reflect the availability of PTEs to a certain extent. For instance, if PTEs in soil exist solely in the fraction of F1, the M_R_ value is maximum, which is 1. Conversely, if PTEs exist only in the fraction of F5, the M_R_ value is minimum, which is 0.04. The average single-element M_R_ value of PTEs around the smelter was higher for Cd (0.59) than for Cu (0.49), indicating that the potential risk of Cd was greater. This approach enabled an accurate and digital assessment of the ecological risk under the continuous external pollution to the surrounding area, while reflecting the geochemical behavior of PTEs in the soil.

## 5. Conclusions

(1) The concentrations of Cu and Cd in the soil surrounding the copper smelter far exceeded the risk screening values of the soil environmental quality standard (GB15618–2018). The concentration of Cu ranged from 97.47 to 1294.63 mg·kg^−1^ with a CV of 0.95, indicating moderate variation, while Cd ranged from 0.14 to 9.06 mg·kg^−1^ with a CV of 1.68, indicating strong variation. Moreover, concentrations of Cu and Cd were found to be continuously accumulating.

(2) The spatial distribution characteristics of all fractions of Cu and Cd were not entirely consistent with the total concentration. For Cu, the highest points of pollution with different orientations appeared in fractions F5. For Cd, the spatial distributions of fractions F1, F3, and F4 were quite distinct from that of Cd-T. Correlation analysis results indicated that soil physicochemical properties, particularly iron oxides, played a crucial role in the migration and transformation of Cu and Cd.

(3) The heterogeneity distribution of Cu and Cd pollution was significant, with Cu accumulation being more severe in the study area and mainly resulting from the production activities of the smelter. Nonetheless, the results of M_R_ analysis emphasized the influence of active fractions of Cu and Cd, with Cd exhibiting a higher value of M_R_ and posing a greater potential risk to the environment.

## Figures and Tables

**Table 1:** Outline of the sequential extraction procedure for Cu and Cd [16].

Fraction	Extractants	Extraction Procedures
exchangeable and carbonate fraction (F1)	16 mL NaAc–HAc (1 M)	25 °C, 6 h shaking
readily reducible iron and manganese oxide fraction (F2)	20 mL NH_2_OH–HCl (0.04 M)	96 °C, 6 h shaking in the dark
organic fraction (F3)	3 mL HNO_3_ (0.02 M) + 5 mL H_2_O_2_ (30%), pH = 2; 5 mL 3.2 M CH_3_COONH_4_	5 h in a water basin at 85 °C; cool, add CH_3_COONH_4_ and shake for 30 min, 25 °C
crystalline iron oxide fraction (F4)	10 mL DCB + Na_2_S_2_O_4_	15 min in a water basin at 80 °C, repeat twice
residual fraction (F5)	2 mL HCl/6 mL HNO_3_/2 mL HF	digestion

**Table 2:** Classification of the geo-accumulation index [28].

Class	Index	Pollution Level
0	I_geo_ ≤ 0	Unpolluted
1	0 < I_geo_ < 1	Slightly polluted
2	1 < I_geo_ < 2	Moderately polluted
3	2 < I_geo_ < 3	Moderately to heavily polluted
4	3 < I_geo_ < 4	Heavily polluted
5	4 < I_geo_ < 5	Heavily to extremely polluted
6	I_geo_ > 5	Extremely polluted

**Table 3:** Soil characteristics and PTE concentrations around the copper smelter.

Items	Maximum	Minimum	Mean	SD	Skewness	Kurt.	CV	K–S Test
pH	5.16	4.04	4.78	0.29	−1.53	2.24	0.06	Non-normal
SOM (%)	4.43	1.09	2.27	0.67	1.84	6.48	0.30	Approximately normal
AP (mg·kg^−1^)	123.29	0.75	31.17	30.71	2.18	4.66	0.99	Approximately log-normal
Fe-T (g·kg^−1^)	64.34	10.37	23.74	15.37	1.82	2.33	0.65	Non-normal
Fe_d_ (g·kg^−1^)	31.47	4.87	9.95	7.01	2.08	4.43	0.70	Non-normal
Fe_o_ (g·kg^−1^)	22.64	0.79	4.73	5.43	2.52	6.79	1.15	Non-normal
Cu (mg·kg^−1^)	1294.63	97.47	317.70	301.13	2.39	6.29	0.95	Approximately log-normal
Cd (mg·kg^−1^)	9.06	0.14	1.14	2.04	4.09	17.09	1.79	Approximately log-normal

Note: CV represents the coefficient of variation; SD represents standard deviation; K–S test represents the Kolmogorov–Smirnov test.

## Data Availability

The authors declare that data supporting the findings of this study are available within the article.

## Supplementary Materials

The following supporting information can be downloaded at: https://www.mdpi.com/article/10.3390/toxics11080647/s1, Table S1: The background value of soil elements and the screening value of soil pollution risk of agricultural land locally; Table S2: The distribution statistics of each form of Cu and Cd.
