# Supplementary material for: Spatial Differentiation Characteristics and Evaluation of Cu and Cd in Paddy Soil around a Copper Smelter
Source: Toxics. 2023 Jul 26;11(8):647. doi: 10.3390/toxics11080647 (PMC10457998; doi:10.3390/toxics11080647)
Supplement: Supplementary file 1 [file toxics-11-00647-s001.zip › toxics-2488198-supplementary.pdf]

Table S1. The background value of soil elements and the screening value of soil pollution risk of agricultural land locally.

Element	Cu	Cd	Pb	Zn	As	Hg
SBV ($mg·kg^{-1}$)	20.30	0.11	32.30	69.40	14.90	0.08
RSV (soil pH ＜ 5.5) ($mg·kg^{-1}$)	50	0.30	250	200	30	0.30

Note: SBV represents soil background values; RSV represents risk screening values according to Chinese soil environmental quality standards (GB 15618-2018).

Table S2. The distribution statistics of each form of Cu and Cd.

PTEs	Forms	Minimum	Maximum	Mean	SD	CV
Cu ($mg·kg^{-1}$)	F1	19.39	326.72	80.79	83.53	1.03
	F2	12.06	261.74	70.45	67.56	0.96
	F3	13.80	315.13	71.01	67.73	0.95
	F4	5.96	137.64	20.61	29.77	1.44
	F5	10.70	282.82	75.96	78.45	1.03
Cd ($mg·kg^{-1}$)	F1	0.03	1.64	0.43	0.37	0.86
	F2	0.01	0.77	0.10	0.18	1.84
	F3	0.00	0.07	0.02	0.02	1.13
	F4	0.00	0.13	0.03	0.03	1.14
	F5	0.04	6.61	0.57	1.51	2.65
